# Supplementary material for: The Effects of Comprehensive Sexual and Reproductive Health/Family Planning Intervention Based on Knowledge, Attitudes, and Practices Among the Domestic Migrant Population of Reproductive Age in China: A Randomized Community Study
Source: Int J Environ Res Public Health. 2020 Mar 21;17(6):2093. doi: 10.3390/ijerph17062093 (PMC7142769; doi:10.3390/ijerph17062093)
Supplement: Supplementary file 1 [file ijerph-17-02093-s001.pdf]

**The Methodology of multivariate probit models**

In some study, researchers need to set a threshold to reach an object. For example, in our study, we analyzed the net effect of the intervention on which contraceptive methods the participants were using. First, we needed the participants who had sex excluding those who had no sex. Second, we needed the participants who were in contraception among those who had sex. Third, we analyzed the net effect of the intervention on which contraceptive methods the participants were using under the two conditions given above. However, this might incur the selection bias.

The methodology is as followed.

We set the selection model as $y_i^{select}=x_i\beta+u_1$, $u_1$~N (0, 1), the latent model as $s_i^*=z_i\delta+u_{2i}$, $u_2$~N (0, 1) and the response model as $y_i^{probit}=s_i^*>0= z_i\delta+u_{2i}$, where $s_i^*$ is a unobserved expected utility[1]. If $s_i^*>0$, $s_i=1$; $s_i^*<0$, $s_i=0$, where $s_i$ is a selection indicator. If $s_i=1$, the participant was included in this model, conversely, the participant was dropped from the data.

E ($u_{1i}|x_i$, $s_i$) = E ($u_{1i}|x_i$, $z_i\delta+u_{2}i>0$) = E ($u_{1i}|x_i$, $u_2i >-z_i\delta$), which means $u_{1i}$ is correlated with $s_i$ if $u_{1i}$ is correlated with $u_{2i}$. Therefore, we just need to test the assumption $\varrho$=corr($u_1$, $u_2$)=0, the selection bias can be found whether exists or not.

For the bivariate probit model, suppose the latent model as $s_i^*=z_i\delta+u_i$, i=1,2. We have

$$y_1=\begin{cases}0 & s_1^* = 0\\ 1 & s_1^* > 0\end{cases} \quad (1)$$

If $y_1=1$ , then $$y_2=\begin{cases}0 & s_2^* \leqq 0\\ 1 & s_2^* > 0\end{cases} \quad (2)$$

Then P($y_1$=0) = $1-\Phi(x_1\beta_1)$; P ($y_1$=1, $y_2$=0) = $\Phi(x_1\beta_1)-\Phi_2(x_1\beta_1, x_2\beta_2, \rho_{12})$; P ($y_1$=1, $y_2$=1) = $\Phi_2(x_1\beta_1, x_2\beta_2, \rho_{12})$

The likelihood function is given by

L ($\tilde{\beta}|\tilde{y},\tilde{x}$) = P ($y_2|y_1$, $x_2$) P ($y_1|x_1$), $\tilde{\beta}$= ($\beta_1$, $\beta_2$), $\tilde{y}$= ($y_1$ $y_2$), $\tilde{x}$= ($x_1$ $x_2$)

The log likelihood function is given by

$$\ln L= \sum_{i=1}^{N}\{y_{i1}y_{i2}\ln\Phi_2(x_1\beta_1, x_2\beta_2, \rho_{12}) + y_{i1}(1-y_{i2})ln[\Phi(x_1\beta_1, \rho_{12}) - \Phi_2(x_1\beta_1, x_2\beta_2, \rho_{12})] + (1-y_{i1})\ln[1-\Phi(x_1\beta_1)]\}$$

Where Φ (.) is the standard normal cumulative distribution function, $\Phi_2$ (.) is the bivariate standard normal cumulative distribution function with the correlation coefficient $\rho_{12} =$

$Cov[u_1u_2|x_1x_2]$.

For the trivariate probit model, suppose the latent model as $s_i$*=$z_i$δ+$u_i$, u~N(0, 1), i=1, 2, 3. We have $y_1=\begin{cases}0 \; s_1^* = 0\\ 1 \; s_1^* > 0\end{cases}$ (1)

If $y_1$=1, then $y_2=\begin{cases}0 \; s_2^* \leqq 0\\ 1 \; s_2^* > 0\end{cases}$ (2)

If $y_1$=1 and $y_2$=1, then $y_3=\begin{cases}0 \; s_3^* \leqq 0\\ 1 \; s_3^* > 0\end{cases}$ (3)

Then P($y_1$=0) $= 1 - \Phi(x_1\beta_1)$; P ($y_1$=1, $y_2$=0) = $\Phi(x_1\beta_1) - \Phi_2(x_1\beta_1, x_2\beta_2, \rho_{12})$;P ($y_1$=1, $y_2$=1, $y_3$=0) = $\Phi_2(x_1\beta_1, x_2\beta_2, \rho_{12}) - \Phi_3(x_1\beta_1, x_2\beta_2, x_3\beta_3, \rho_{12}, \rho_{13}, \rho_{23},)$ P ($y_1$=1, $y_2$=1, $y_3$=1) = $\Phi_3(x_1\beta_1, x_2\beta_2, x_3\beta_3, \rho_{12}, \rho_{13}, \rho_{23},)$

The likelihood function is given by

L $(\tilde{\beta}|\tilde{y}, \tilde{x})$ = P ($y_3$|$y_2$, $y_1$, $x_3$) P ($y_2$|$y_1$, $x_2$) P ($y_1$|$x_1$), $\tilde{\beta}$= ($\beta_1$, $\beta_2$, $\beta_3$), $\tilde{y}$= ($y_1$ $y_2$ $y_3$), $\tilde{x}$= ($x_1$ $x_2$ $x_3$).

The log likelihood function is given by

lnL= $\sum_{i=1}^{N}\{y_{i1}y_{i2}y_{i3}\ln\Phi_3(x_1\beta_1, x_2\beta_2, x_3\beta_3, \rho_{12}, \rho_{13}, \rho_{23}) + y_{i1}y_{i2}(1-y_{i3})ln[\Phi_2(x_1\beta_1, x_2\beta_2, \rho_{12}) - \Phi_3(x_1\beta_1, x_2\beta_2, x_3\beta_3, \rho_{12}, \rho_{13}, \rho_{23})] + y_{i1}(1-y_{i2})[\Phi(x_1\beta_1) - \Phi_2(x_1\beta_1, x_2\beta_2, \rho_{12})] + (1 - y_{i1})\ln[1-\Phi(x_1\beta_1)]\}$.

Where $\Phi_3$ (.) is the trivariate standard normal cumulative distribution function with correlation coefficients $\rho_{12} = Cov[u_1u_2|x_1x_2]$, $\rho_{13} = Cov[u_1u_3|x_1x_3]$, $\rho_{12} = Cov[u_2u_3|x_2x_3]$.

Similarly, we can get the log likelihood function of the Quavariate model, which is given by

lnL= $\sum_{i=1}^{N}\{y_{i1}y_{i2}y_{i3}y_{i4}\ln\Phi_4(x_1\beta_1, x_2\beta_2, x_3\beta_3, x_4\beta_4, \rho_{12}, \rho_{13}, \rho_{23}, \rho_{14}, \rho_{24}, \rho_{34}) + y_{i1}y_{i2}y_{i3}(1 - y_{i4})ln[\Phi_3(x_1\beta_1, x_2\beta_2, x_3\beta_3, \rho_{12}, \rho_{13}, \rho_{23}) - \Phi_4(x_1\beta_1, x_2\beta_2, x_3\beta_3, x_4\beta_4, \rho_{12}, \rho_{13}, \rho_{23}, \rho_{14}, \rho_{24}, \rho_{34})] + y_{i1}y_{i2}(1-y_{i3})[\Phi_2(x_1\beta_1, x_2\beta_2, \rho_{12}) - \Phi_3(x_1\beta_1, x_2\beta_2, x_3\beta_3, \rho_{12}, \rho_{13}, \rho_{23})] + y_{i1}(1 - y_{i2})\ln[\Phi(x_1\beta_1) - \Phi_2(x_1\beta_1, x_2\beta_2, \rho_{12})] + (1-y_{i1})\ln[1-\Phi(x_1\beta_1)]\}$

Where $\Phi_4$ (.) is the Quavariate standard normal cumulative distribution function with correlation coefficients $\rho_{12} = Cov[u_1u_2|x_1x_2]$, $\rho_{13} = Cov[u_1u_3|x_1x_3]$, $\rho_{23} = Cov[u_2u_3|x_2x_3]$, $\rho_{14} = Cov[u_1u_4|x_1x_4]$, $\rho_{24} = Cov[u_2u_4|x_2x_4]$, $\rho_{34} = Cov[u_3u_4|x_3x_4]$.

Assuming that $s_1^*$ and $u_i$ are normally distributed, full maximum likelihood estimation requires a multivariate probit model, which is consistent and asymptotically efficient[1]. The parameter estimation in such models was done with the GHK algorithm[2].

**Reference**

1. Rosenman R, Mandal B, Tennekoon V, Hill LG. Estimating treatment effectiveness with sample selection. Washington State University https://coreacuk/download/pdf/6836288pdf. 2010.

2. Roodman D. Estimating fully observed recursive mixed-process models with cmp. 2009.
